# Design and Analysis of a Novel Bionic Tensegrity Robotic Fish with a Continuum Body

**DOI:** 10.3390/biomimetics9010019

**Published:** 2024-01-02

**Authors:** Di Chen, Bo Wang, Yan Xiong, Jie Zhang, Ru Tong, Yan Meng, Junzhi Yu

**Affiliations:** 1 State Key Laboratory for Turbulence and Complex Systems, Department of Advanced Manufacturing and Robotics, College of Engineering, Peking University, Beijing 100871, China; di.chen@pku.edu.cn (D.C.); bowang23@stu.pku.edu.cn (B.W.); x.yan@stu.pku.edu.cn (Y.X.); yan.meng@pku.edu.cn (Y.M.); 2 School of Aeronautics and Astronautics, Sun Yat-sen University, Shenzhen 518107, China; zhangj696@mail2.sysu.edu.cn; 3 Laboratory of Cognitive and Decision Intelligence for Complex System, Institute of Automation, Chinese Academy of Sciences, Beijing 100190, China; tongru2019@ia.ac.cn; 4 Science and Technology on Integrated Information System Laboratory, Institute of Software, Chinese Academy of Sciences, Beijing 100190, China

**Keywords:** bionic robotic fish, tensegrity structure, continuum body, stiffness variation, swimming performance

## Abstract

Biological fish exhibit remarkable adaptability and exceptional swimming performance through their powerful and flexible bodies. Therefore, designing a continuum flexible body is significantly important for the development of a robotic fish. However, it is still challenging to replicate these functions of a biological body due to the limitations of actuation and material. In this paper, based on a tensegrity structure, we propose a bionic design scheme for a continuum robotic fish body with a property of stiffness variation. Its detailed structures and actuation principles are also presented. A mathematical model was established to analyze the bending characteristics of the tensegrity structure, which demonstrates the feasibility of mimicking the fish-like oscillation propulsion. Additionally, the stiffness variation mechanism is also exhibited experimentally to validate the effectiveness of the designed tensegrity fish body. Finally, a novel bionic robotic fish design scheme is proposed, integrating an electronic module-equipped fish head, a tensegrity body, and a flexible tail with a caudal fin. Subsequently, a prototype was developed. Extensive experiments were conducted to explore how control parameters and stiffness variation influence swimming velocity and turning performance. The obtained results reveal that the oscillation amplitude, frequency, and stiffness variation of the tensegrity robotic fish play crucial roles in swimming motions. With the stiffness variation, the developed tensegrity robotic fish achieves a maximum swimming velocity of 295 mm/s (0.84 body length per second, BL/s). Moreover, the bionic tensegrity robotic fish also performs a steering motion with a minimum turning radius of 230 mm (0.68 BL) and an angular velocity of 46.6°/s. The conducted studies will shed light on the novel design of a continuum robotic fish equipped with stiffness variation mechanisms.

## 1. Introduction

In nature, biological fish have evolved into versatile swimmers with excellent performance. A large number of features, such as fast speed, agility, high efficiency, and strong adaptability in complex aquatic environments, are extremely fascinating for researchers [1]. These astonishing capabilities mainly derive from their unique physical structures including morphological characteristics and flexible bodies with powerful musculature, which offer extensive inspiration for the ideal underwater platform development to perform aquatic animal supervision [2], ocean exploration [3,4], marine environment monitoring and protection [5], disaster rescue, and so on. The majority of biological fish adopt the BCF (body and/or caudal fin) pattern as their primary propulsion mode to realize high-performance locomotion [6]. These fish usually bend their flexible body with continuous muscles to obtain reactive force from water. More importantly, body stiffness can be actively modulated to adapt to different fluid environments to show outstanding swimming motion [7]. Many investigations have indicated that the tunable stiffness mechanism is of great significance for the design of bionic underwater robots with high performance [8,9,10].

In the past few decades, various bionic robotic fish with BCF mode have been developed [11,12]. From the perspective of bioinspired design, these robots can be roughly classified into the discrete mechanism with multiple joints and the continuous compliant mechanism [13,14]. Specifically, traditional robotic fish are typically designed with a discrete body structure connected by a series of joints to imitate flexible undulation propulsion. However, this multi-joint design scheme often necessitates numerous motors and precise joint angle controls, resulting in high costs, challenging control requirements, and low efficiency. Recognizing the significant importance of emulating biological locomotion through the exploration of biological fish’s propulsion mechanism [15,16,17], researchers have proposed incorporating flexible components into multi-joint robotic fish to enhance performance. For example, Chen et al. integrated a compliant joint into a multi-joint robotic fish and explored the influence of control parameters and joint stiffness to improve swimming performance [18]. White et al. experimentally explored the body flexibility configurations with different numbers of compliant joints and gained a high speed of 4.6 BL/s at a frequency of 8.0 Hz [19]. Zou et al. developed a tail with two flexible joints, and a dynamic model-based optimization method was proposed to optimize the stiffness distribution [20]. However, the optimal stiffness is dependent on various variables and a single-stiffness flexible component may not always meet the requirements for performance enhancement under all conditions [8]. Therefore, several variable stiffness mechanisms in different fields have been proposed [21,22,23,24]. Nevertheless, the integration of complex structures poses challenges. Only a limited number of robots are capable of achieving variable stiffness. For instance, Zhong et al. developed a robotic fish with an adjustable stiffness mechanism where a servo motor is employed to modify the pretension force of a spring connected to a passive joint [9].

In the case of continuum robotic fish, an elastic spine is commonly utilized to constitute a flexible posterior body [25,26]. This design requires only one motor and the elastic spine serves as a compliant passive component. As such, these robots are simple in design, easy to control, and cost-effective. The passive mechanism is determined by the stiffness of elastic components which can be optimized for performance improvement. Li et al. developed a soft robotic fish with a variable stiffness mechanism, by adjusting the stiffness, a maximum speed of 0.54 BL/s (body length per second) was achieved [27]. A tethered soft robotic fish with pneumatic actuators and a flexible foil was developed. The body stiffness can be adjusted by changing the pressure, and a maximum speed of 130 mm/s was achieved [28]. Qiu et al. designed a tendon-driven structure and a variable passive caudal joint for a robotic fish and emphatically analyzed the effects of the caudal joint’s stiffness on performance [29]. Kwak et al. proposed a stiffness-adjustable paddle with a sliding laminate-based method and integrated it into an untethered swimming robot. By displacing a flexible sheet with rigid elements to offset the alignment state with the opposite layer, two different stiffness states can be achieved [30]. However, these proposed variable stiffness mechanisms usually require an extra structure increasing system complexity.

The tensegrity structure composed of rigid elements connected by a network of elastic cables to maintain stability has demonstrated great potential in realizing the variable stiffness mechanism of a body. With the pre-stretched elastic cables, the tensegrity system can perform a compliant characteristic. Adding extra wire-driven actuation, some variable stiffness mechanisms have been proposed in building spine structures or robot arms [31,32,33]. For example, by actively adjusting a ball-joint constraint between adjacent vertebrae, Zappetti et al. proposed a variable stiffness tensegrity spine with three stiffness modes [31]. The related works also provide some new insights into the fish robot design. Bliss et al. proposed a tensegrity structure for underwater propulsion and experimentally explored the effects of central pattern generator control [34]. Shintake et al. developed a fish-like robot with a simple tensegrity structure. By tuning the elastic cables, the stiffness of the tensegrity structure can be changed, and a maximum speed of 230 mm/s (0.58 BL/s) was achieved [35]. Chen et al. developed a fish robot with variable-stiffness tensegrity joints. By changing the stiffness distribution, the swimming performance can be enhanced tremendously, and a maximum speed of 0.87 BL/s was achieved [36]. These works have demonstrated the potential of stiffness variation in enhancing performance, yet the use of pre-programmed stiffness remains prevalent and the maneuverability aspect is often less considered.

As mentioned above, the robotic fish with discrete mechanisms usually integrate flexible components with only a single stiffness [18,19,20], the continuum robotic fish with stiffness variation mechanism commonly employ an extra structure [27,28,29,30], and the latest emerged robotic fish with tensegrity structure also adopt the pre-programmed stiffness and rarely pay attention to the maneuverability [34,35,36]. Toward these problems, there are two primary contributions of this study: First, based on a novel modular tensegrity structure, we propose a bionic flexible and continuum fish body that can realize the fish-like oscillation and online stiffness variation simultaneously. Furthermore, a mathematical model of the adopted tensegrity structure was built to explore its bending property. The characteristics of the tensegrity body in fish-like oscillation motion and stiffness variation were also experimentally analyzed. Second, based on the validated continuum tensegrity body, we developed a bionic robotic fish featuring a wire-driven mode and experimentally explored the influences of control parameters and stiffness variation on swimming velocity and maneuverability. Results have demonstrated the effectiveness of our proposed tensegrity robotic fish with stiffness variation in performance improvement, offering some new insights into the underwater robot design.

The remaining sections of this paper are organized as follows: In Section 2, we give details on the continuum body with the tensegrity structure design and its analyses on bending, oscillation motion, and stiffness variation. Section 3 presents the overall system design and fabrication of the continuum tensegrity robotic fish. The systematical experiments with different control parameters and stiffness variations were conducted in Section 4. Section 5 shows some detailed discussions and some conclusions and future works are finally summarized in Section 6.

## 2. Structure Design of Bionic Tensegrity-Based Robotic Fish Body

### 2.1. Design of the Tensegrity Fish Body

**Figure 1:**
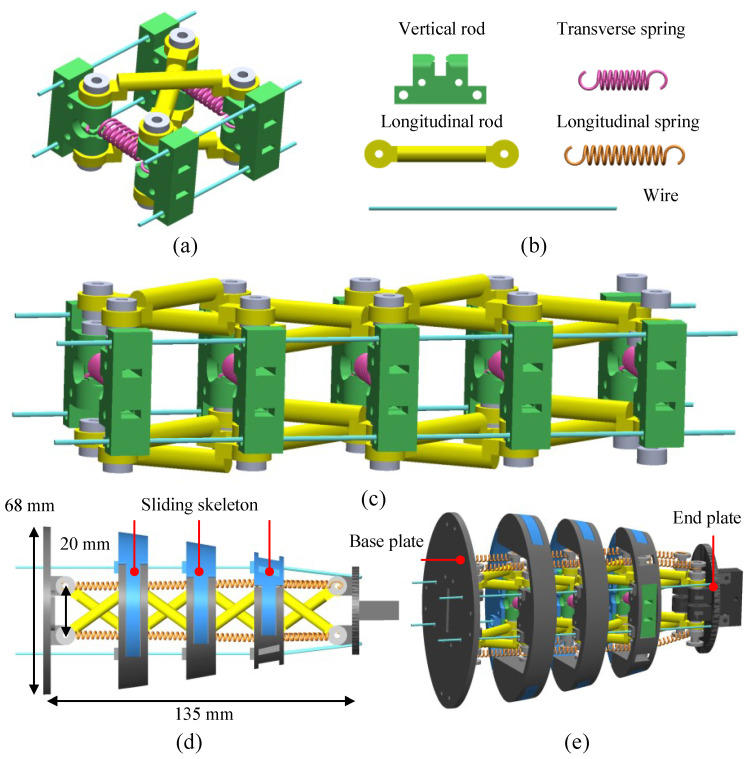
Illustration of a bionic tensegrity body. (**a**) Modular tensegrity unit. (**b**) Tensegrity components. (**c**) Tensegrity structure with four tensegrity units. (**d**) Top view of the tensegrity robotic fish body. (**e**) Side view of the tensegrity robotic fish body.

In nature, the biological fish body exhibits powerful and flexible properties with the integration of tissues, bones, and muscles. However, replicating these actuation capabilities using traditional robotic fish design approaches poses significant challenges. Tensegrity systems have demonstrated attractive characteristics in high strength-to-mass ratios, flexibility, and tunable stiffness [34]. Therefore, in this study, we conceptualize the fish’s body architecture as a tensegrity system and utilize it as the backbone for our robotic fish’s posterior body. Figure 1 illustrates the adopted modular tensegrity structure and the design of the tensegrity robotic fish body. As depicted in Figure 1a, a single tensegrity unit consists of two rectangle-like layers that are cross-linked by two rigid longitudinal rods and two pre-tensioned transverse springs. Four vertical rods with equal lengths are divided into two groups and connected to the longitudinal rods via rotational hinges. By serially integrating these modular tensegrity units, a continuous tensegrity structure can be easily constructed. Figure 1c demonstrates a four-unit configuration of our designed tensegrity structure.

As presented in Figure 1d,e, a tensegrity continuum fish body with a serial structure of modular tensegrity units is proposed. The base plate connecting the first modular tensegrity unit will be fixed at the head of a robotic fish, while the end plate connecting the last modular tensegrity unit will be linked with a fishtail. To maintain a streamlined body shape, we have designed skeletons inspired by fish profiles. When oscillating in water, hydrodynamic forces act on the structure, potentially causing unexpected deformation. Therefore, to better mimic fish-like oscillation propulsion and enhance stability during pretension adjustment, two additional designs are proposed. Firstly, extra pre-tensioned longitudinal springs are added to the vertical rods in each group, which helps to preserve body shape. Secondly, when the wires are driven in the longitudinal direction simultaneously, the tensegrity body contracts lengthwise and extends transversely. To enhance the stability of body shape, the skeletons with sliding structures are designed. As mentioned above, both transverse springs and longitudinal springs determine the stiffness of our tensegrity body; their physical properties are summarized in Table 1.

In this study, we employ the wire-drive mode to actuate the tensegrity body and replicate fish-like oscillation propulsion motion. Specifically, two sets of wires are utilized to independently drive each side of the tensegrity body. In particular, two servo motors with wire wheels are adapted to drive the wires, which can further improve the operation modes of the robotic fish body. To elucidate the actuation principle, we define $L_{1}$ as the length of the wire on the right side and $L_{2}$ as that on the left side. The variations in their lengths are denoted by $\Delta L_{1}$ and $\Delta L_{2}$ , respectively. Similar to other wire-driven robotic fish, when there is a difference between $\Delta L_{1}$ and $\Delta L_{2}$ , bending motion is generated in the robot body. By controlling the rotation angles of two servo motors, the tensegrity body can easily perform periodic fish-like oscillation motion to realize the straight swimming motion or steering motion. For a biological fish, the stiffness modulation of the body is commonly achieved by the contraction and relaxation of muscles. Inspired by this phenomenon and the deployable characteristic of the tensegrity system, our tensegrity body can also realize stiffness variation with the contraction and release of wires. Concretely, when $\Delta L_{1}=\Delta L_{2}$ , the body will be shortened synchronously. Consequently, longitudinal springs compress while transverse springs stretch accordingly, similar to the action of muscles. With appropriate adjustment of the spring’s pretension, stiffness variation within our proposed tensegrity body can also be realized.

### 2.2. Bending Analysis of the Tensegrity Structure

In this study, the tensegrity unit is actuated by two sets of wires. By adjusting the wire lengths on both sides of the tensegrity unit, its shape can be altered accordingly. To accurately depict the correlation between bending angle and length variation of the actuated wires, we have developed a mathematical model for analyzing the behavior of tensegrity structures.

**Figure 2:**
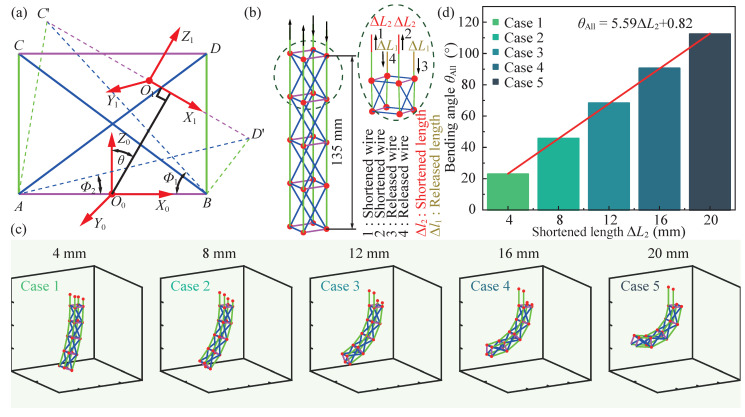
Bending analysis of the tensegrity structure with a mathematical model. (**a**) Illustration of the simplified tensegrity unit model. (**b**) Wire-driven mode of the tensegrity structure. (**c**) Numerical simulation results of the tensegrity structure’s bending motion under different lengths of wires. (**d**) Bending angles of the tensegrity structure’s deformation under different lengths of wires.

As illustrated in Figure 2a, to clarify the description of the model, some coordinate frames and notations are defined. The tensegrity unit is simplified as a quadrilateral with two diagonal lines. The quadrilateral ABCD is the initial state of a tensegrity unit. Sideline AC and BD indicate the two driven wires. The quadrilateral $ABC^{\prime }D^{\prime }$ is the deformed state after the two groups of wires are actuated. The diagonal lines of these quadrilaterals denote the longitudinal roads whose lengths remain constants. Namely, $AD=BC=AD^{\prime }=BC^{\prime }=b$ . The base frame and tensegrity unit frames are defined as $C_{0}=O_{0}-X_{0}Y_{0}Z_{0}$ and $C_{i}=O_{i}-X_{i}Y_{i}Z_{i},\left(i=1,2,\ldots ,N\right)$ , respectively. As an example, the frame $C_{1}=O_{1}-X_{1}Y_{1}Z_{1}$ is shown in Figure 2a. Planes $X_{0}O_{0}Z_{0}$ and $X_{1}O_{1}Z_{1}$ all coincide with the planes of these quadrilaterals. Origins $O_{0}$ and $O_{1}$ are located at the midpoints of AB and $C^{\prime }D^{\prime }$ , respectively. $\Phi_{1}$ and $\Phi_{2}$ denote the angle between AB and $BC^{\prime }$ and the angle between AB and $AD^{\prime }$ , respectively. θ represents the rotation angle of the tensegrity unit. According to the geometric relationships between quadrilateral ABCD and $ABC^{\prime }D^{\prime }$ , the lengths of $AC^{\prime }$ and $BD^{\prime }$ can be calculated in the following form:

(1) $$\left\{\begin{array}{c} {\left(AC^{\prime }\right)}^{2}=AB^{2}+{\left(BC^{\prime }\right)}^{2}-2AB\times BC^{\prime }\cos\Phi_{1}\\ {\left(BD^{\prime }\right)}^{2}=AB^{2}+{\left(AD^{\prime }\right)}^{2}-2AB\times AD^{\prime }\cos\Phi_{2} \end{array}\right..$$

Define the length of AB=a and $AC=BD=L_{c}$ . Let the length variation of two groups of wires in a tensegrity unit as $\Delta l_{1}$ and $\Delta l_{2}$ , respectively. Then, the length of $AC^{\prime }$ and $BD^{\prime }$ can be calculated as $L_{c}+\Delta l_{1}$ and $L_{c}-\Delta l_{2}$ . Take these expressions into Equation (1), the angles of two longitudinal rods can be calculated in the following form:

(2) $$\left\{\begin{array}{c} \;\Phi_{1}=\arccos\left(\frac{a^{2}+b^{2}-L_{c}^{2}-\Delta l_{1}^{2}-2L_{c}l_{1}}{2ab}\right)\\ \;\Phi_{2}=\arccos\left(\frac{a^{2}+b^{2}-L_{c}^{2}-\Delta l_{2}^{2}+2L_{c}l_{2}}{2ab}\right) \end{array}\right..$$

Then, the coordinates of points C’ , $D^{\prime }$ , and origin $O_{1}$ in frame $C_{0}$ can be expressed as follows:

(3) $$\left\{\begin{array}{c} x_{C^{\prime }}=\frac{a}{2}-b\cos\Phi_{1}\\ \;z_{C^{\prime }}=b\sin\Phi_{1} \end{array}\right.,\left\{\begin{array}{c} x_{D^{\prime }}=b\cos\Phi_{2}-\frac{a}{2}\\ \;z_{D^{\prime }}=b\sin\Phi_{2} \end{array}\right.,\left\{\begin{array}{c} \;x_{O_{1}}=\frac{x_{C^{\prime }}+x_{D^{\prime }}}{2}\\ \;z_{O_{1}}=\frac{z_{C^{\prime }}+z_{D^{\prime }}}{2} \end{array}\right..$$

Finally, the rotation angle of a tensegrity unit can be calculated as below.

(4) $$\theta =\arctan k_{C^{\prime }D^{\prime }}$$

where $k_{C^{\prime }D^{\prime }}=\left(\frac{z_{D^{\prime }}-z_{C^{\prime }}}{x_{D^{\prime }}-x_{C^{\prime }}}\right)$ is the slope of line $C^{\prime }D^{\prime }$ in frame $O_{0}-X_{0}Y_{0}Z_{0}$ .

Similarly, according to the derivation mentioned above, we can also calculate the rotation angle $\theta_{i}$ and the coordinates of the *i*th tensegrity unit. As for a tensegrity structure with *N* units, the positions of a point in *i*th tensegrity unit can be expressed in the base frame $C_{0}$ by a series of coordinate transformations. The transformation matrix between frame $C_{i}$ and frame $C_{i-1}$ can be expressed as:

(5) $${\;}^{i-1}T_{i}=\left[\begin{array}{cc} {\;}^{i-1}R_{i} & {\;}^{i-1}P_{i}\\ 0 & 1 \end{array}\right],{\;}^{i-1}R_{i}=\left[\begin{array}{ccc} \cos\theta_{i-1} & 0 & \sin\theta_{i-1}\\ 0 & 1 & 1\\ -\sin\theta_{i-1} & 0 & \cos\theta_{i-1} \end{array}\right.,{\;}^{i-1}P_{i}=\left[\begin{array}{c} x_{o_{i}}\\ 0\\ z_{o_{i}} \end{array}\right],$$

where ${\;}^{i-1}R_{i}$ is the rotation matrix, ${\;}^{i-1}P_{i}$ represents the position vector of origin $O_{i}$ in frame $C_{i-1}$ , $\theta_{i-1}$ means the rotation angle of $i_{i-1}$ th tensegrity unit, and $x_{O_{i}}$ and $z_{O_{i}}$ denote the coordinates of origin $O_{i}$ in frame $C_{i-1}$ .

Then, the position vector in *i*th tensegrity unit can be calculated in the following form:

(6) $$P_{i}={\;}^{0}T_{i}{\;}^{i}P_{i},\left(i=0,1,2,\ldots ,N\right),$$

where ${\;}^{0}T_{i}={\;}^{0}T_{1}{\;}^{1}T_{2}\ldots {\;}^{i-1}T_{i}$ , ${\;}^{i}P_{i}$ means the position vector in frame $C_{i}$ .

The length variation of each tensegrity unit is assumed as the same and can be calculated as

(7) $$\left\{\begin{array}{c} \;\Delta l_{1}^{\left(1\right)}=\Delta l_{1}^{\left(2\right)}=\ldots =\Delta l_{1}^{\left(N\right)}=\frac{\Delta L_{1}}{N}\\ \Delta l_{2}^{\left(1\right)}=\Delta l_{2}^{\left(2\right)}=\ldots =\Delta l_{2}^{\left(N\right)}=\frac{\Delta L_{2}}{N} \end{array}\right.,$$

where $\Delta L_{1}$ and $\Delta L_{2}$ are the total length variation of wires on each side, and the bending angle of the tensegrity body can be calculated as

(8) $$\theta_{All}=N\theta .$$

In our design of the flexible continuum body, we employ a series configuration consisting of four tensegrity units, as depicted in Figure 2b. Its desired drive mode is also shown. By selectively shortening and releasing wires on opposite sides, the body exhibits a bending motion resembling that of fish-like oscillation propulsion. In addition, based on the model, we investigate the influence of length variation on the bending behavior of the tensegrity structure. As illustrated in Figure 2c, five cases with different shortened lengths ( $\Delta L_{2}=$ 4 mm, 8 mm, 12 mm, 16 mm, and 20 mm) are considered, and the simulated bending motion of the tensegrity structure with four units are presented visually. The simulation animation is provided in the Supplementary Materials. According to the model, the deformations with bending angles are also estimated and shown in Figure 2d. Notably, our results demonstrate a linear relationship between the bending angle and shortened length $\Delta L_{2}$ . Namely, $\theta_{All}=5.59\Delta L_{2}+0.82$ . This periodic shortening and releasing mechanism enables controlled bending motions in both rightward and leftward directions, imitating a fish-like oscillation propulsion mode.

### 2.3. Analysis of the Tensegrity Body with Stiffness Variation

The rigid rods in a tensegrity structure are interconnected by a network of interconnected elastic elements, ensuring mechanical stability and compliant behavior. By adjusting the pre-stretch length or the elasticity of cables, the variable stiffness of the tensegrity system can be achieved. It is important to note that changing the elasticity typically requires replacing elastic elements, resulting in offline stiffness variation. However, modifying the pre-stretch length can be easily accomplished through an actuation mechanism, allowing for direct changes in stiffness. As for a flexible body with a tensegrity structure, we can achieve the stiffness variation with the two servo motors. When both servo motors rotate at identical angles, contraction occurs symmetrically on both sides of the body. Consequently, this leads to alterations in the pre-tensioning of elastic elements such as transverse springs and longitudinal springs, thereby varying the compliance characteristics of the body. On this basis, two servo motors oscillate periodically to drive the wires, and the body with a different stiffness can perform a fish-like bending motion. With the changed length of the body, its bending characteristic is also changed.

**Figure 3:**
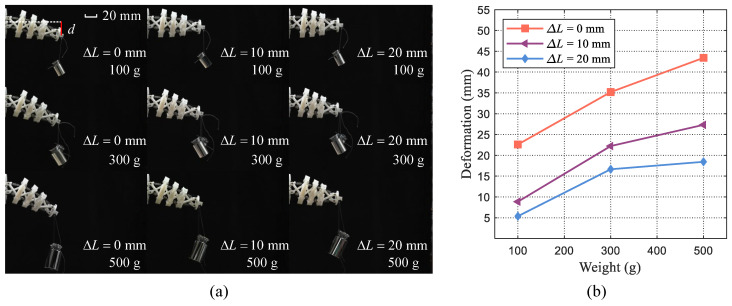
Illustration of the designed tensegrity body’s stiffness variation. (**a**) Experiments of stiffness variation of tensegrity body under different weights and contraction lengths. (**b**) Deformation comparisons of tensegrity body under different loaded weights and contraction lengths.

Firstly, some experiments with different pre-tensions of elastic elements are conducted to exhibit the variation in stiffness properties. Two groups of wires have equal contraction lengths, denoted as $\Delta L_{1}=\Delta L_{2}$ . To simplify the expression, we define ΔL as $\Delta L_{1}=\Delta L_{2}$ . Concretely, the tensegrity body with three contraction lengths ( ΔL = 0 mm, 10 mm, 20 mm) are considered and three kinds of weights (100 g, 300 g, and 500 g) are employed as the load adding to the tensegrity body. The vertical deformation is used as an indicator to quantify the difference in body stiffness. Finally, the tensegrity body deformations under these situations are presented in Figure 3a. It can be observed that as the weight increases, the deformation also increases. Moreover, when applying the same weight load, tensegrity bodies with different contraction lengths exhibit varying degrees of deformation which indicates the different stiffness of the compliant body. This implies that by adjusting the length of driven wires within the body structure, its overall stiffness can be modified accordingly. The deformation values are also measured and shown in Figure 3b. Notably, as for the same load, the deformation of the body with ΔL = 0 mm is larger than that of other situations. And the deformation of the body with ΔL = 20 mm is minimum. With the same load, the large deformation usually indicates the small body stiffness. Despite identical increments in contraction length, the resulting changes in deformation exhibit non-uniform patterns and diminish gradually. This indicates an inherent heterogeneity in stiffness variation. In the case of our designed tensegrity body, a larger contraction length yields a correspondingly smaller deformation value, thereby implying enhanced overall body stiffness.

**Figure 4:**
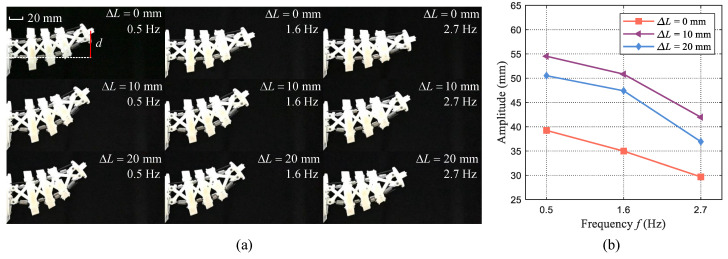
Illustration of the designed tensegrity body’s oscillation amplitude variation. (**a**) Maximum oscillation amplitude of the tensegrity body under different frequencies and contraction lengths. (**b**) Oscillation amplitude comparisons of the tensegrity body under different frequencies and contraction lengths.

In the fish-like oscillation motion, the propulsion is significantly affected by a body’s bending properties. The oscillation amplitude of the body can serve as an indicator to reflect the swimming performance. Generally, a larger oscillation amplitude enables better swimming velocity. However, for our designed tensegrity body, variations in stiffness also result in changes in body length, which complicates the analysis of bending properties. In this subsection, the oscillation amplitude is measured to explore the properties of the designed tensegrity body under different contraction lengths. Concretely, three distinct different actuation frequencies (0.5 Hz, 1.6 Hz, and 2.7 Hz) and three contraction lengths ( ΔL = 0 mm, 10 mm, and 20 mm) are considered to perform the fish-like oscillation motion with wire-driven mode. Two servo motors rotate in a sinusoidal form with an opposite direction. The rotation amplitude of the servo motor is about 65°. The bending motions with maximum amplitudes during tensegrity body oscillations are depicted in Figure 4a. The oscillation amplitude is measured and shown in Figure 4b. The testing videos are also provided in the Supplementary Materials. These results demonstrate that both frequency and contraction length influence changes in the body’s oscillation amplitude. At the same frequency, the oscillation amplitude of the case with a contraction length ΔL = 10 mm is maximum, and the oscillation amplitude of the case with ΔL = 0 mm is the minimum. It should be noted that the oscillation amplitude does not exhibit a linear relationship with the contraction length. Both the body length and the stiffness result in this phenomenon. Additionally, we observe a gradual decrease in oscillation amplitude as the frequency increases. This phenomenon can primarily be attributed to the actuation limitations of the servo motors, which tend to reduce rotation amplitudes at higher frequencies.

## 3. Design and Fabrication of the Bionic Tensegrity Robotic Fish

### 3.1. Mechanical Design of the Tensegrity Robotic Fish

**Figure 5:**
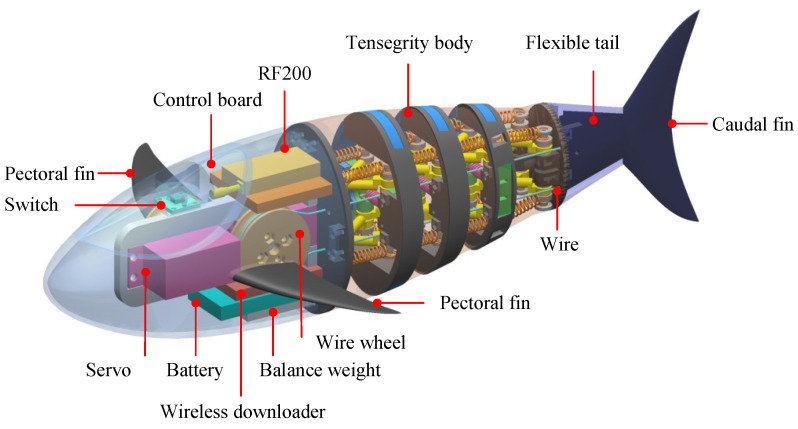
Mechanical design of the bionic tensegrity robotic fish.

By integrating the proposed tensegrity robotic fish body, a novel robotic fish is designed, as illustrated in Figure 5. To reduce the drag during the swimming motion, a body profile with a well-streamlined shape is adopted. The tensegrity robotic fish mainly comprises three parts: a rigid head, a tensegrity body, and a compliant tail with a caudal fin. The head cabin with a pair of pectoral fins is 3D-printed and utilized to contain electronic modules and servo motors. A control board with a communication module is used to receive the commands and generate control signals. Two servo motors are used to actuate the wires on each side of the tensegrity body. Wire wheels with a diameter of 30 mm are employed to amplify wire drive length. Due to space constraints, the pectoral fins remain fixed in position. To improve the propulsion capability, a composite tail including a caudal fin is designed.

In the morphology design of our robotic fish, we follow some basic bionic design principles. The profile of a real fish with some engineering simplifications is directly adopted to design our robotic fish. Similarly, the shapes of a pair of pectoral fins and a caudal fin also come from biological fish and are dealt with by some engineering approaches. For example, the fins are designed with a given chordwise cross-section of a NACA 0018 airfoil. Geometry plays an important role in swimming performance; however, the main goal is to explore the tensegrity stiffness variation mechanism in swimming performance, the optimizations of body geometry and fin shape are not further considered in this paper.

### 3.2. Fabrication of the Tensegrity Robotic Fish

**Figure 6:**
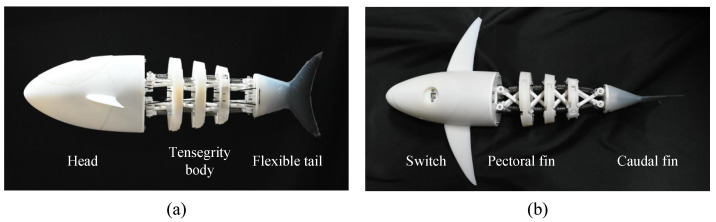
Prototype of the tensegrity robotic fish. (**a**) Side view of the robotic fish. (**b**) Top view of the robotic fish.

With the proposed mechanical design, the robotic fish prototype is developed. As shown in Figure 6. The primary structures including a headshell with pectoral fins and components of tensegrity body are all fabricated using 3D printing technology. Hence, the proposed design scheme offers ease of fabrication at a low cost. As for the flexible tail and caudal fin, a 3D-printed mold is also made and a soft plate constructed from Carbon Fiber Reinforced Composites (CFRP) with a thickness of 0.3 mm is embedded into the middle position. Subsequently, the silica gel is poured into the mold. An elastic and emulsion-made skin with a thickness of 0.03 mm is used to cover the posterior body for waterproofing. Enough extra skin is left, and its effect on stiffness can be neglected. The 3D-printed tensegrity body and head are lightweight. To enhance the stability and maintain an upright posture like a real fish, we added balance weights in suitable positions to achieve a suitable mass distribution. We empirically adjust the posture of our bionic robotic fish in the water by changing the locations and numbers of balance weights. The expected goal is to maintain a little positive buoyancy and an upright posture. Finally, balance weights with a mass of 300 g are adopted. The developed tensegrity robotic fish has a length of 360 mm and a weight of 835 g. The detailed configurations are tabulated in Table 2.

## 4. Experiments and Performance Analysis of the Tensegrity Robotic Fish

### 4.1. Experimental Setup

To explore the swimming performance, we carried out extensive experiments in a water tank with a size of 5 m in length, 4 m in width, and 1.2 m in depth. In addition, a global camera mounted directly above the pool is used to record the swimming motion of our developed tensegrity robotic fish. By analyzing the videos of swimming motion with a customized motion measurement system, both the trajectory and velocity of the swimming motion can be obtained conveniently. In these experiments, the tensegrity robotic fish is attached by red labels, which helps to recognize the robot easily. Even though some balance weights have been added to our robot, a little position buoyancy is left to enhance its stability during the swimming motion.

### 4.2. Swimming Velocity Testing

**Figure 7:**
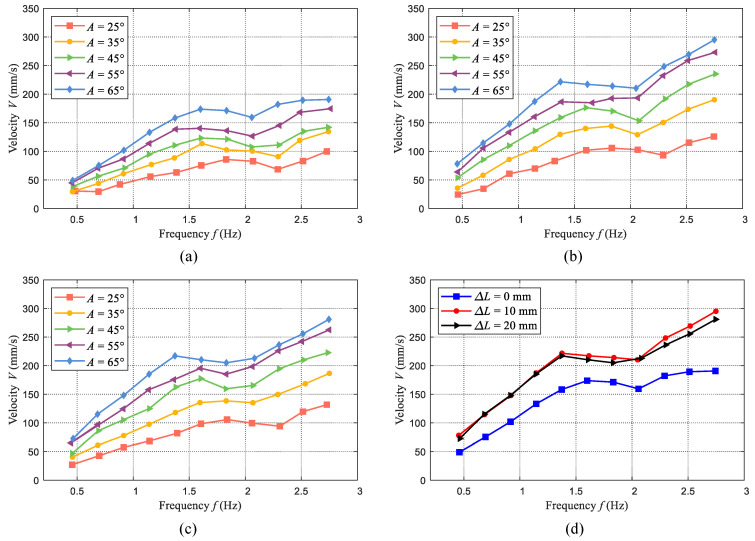
Measured swimming velocity of the designed tensegrity robotic fish versus oscillation frequency and amplitude. (**a**) Swimming velocity with a contraction length of ΔL = 0 mm. (**b**) Swimming velocity with a contraction length of ΔL = 10 mm. (**c**) Swimming velocity with a contraction length of ΔL = 20 mm. (**d**) Swimming velocity comparisons with different contraction lengths ( $A=65^{{}^{\circ}}$ ).

To validate the feasibility of the proposed design of tensegrity robotic fish, swimming motions were performed and the velocities were systemically measured. As for our robotic fish, its swimming velocity is significantly determined by control parameters (frequency and amplitude) and structure parameters (body stiffness). To realize the fish-like oscillation propulsion motion, two servo motors rotate in a sinusoidal form Asin(2πft+φ) . Control parameters include the rotation frequency *f*, amplitude *A*, and phase φ . In the straight swimming motion, the same frequency and amplitude are set to servo motors and their rotation direction is opposite ( φ = 180°). To explore the influence of these parameters on swimming motion, eleven rotation frequencies and five rotation amplitudes ( $A=25^{{}^{\circ}}$ , $35^{{}^{\circ}}$ , $45^{{}^{\circ}}$ , $55^{{}^{\circ}}$ , and $65^{{}^{\circ}}$ ) are adopted. In addition, to exhibit the influences of variable body length of the tensegrity-based robotic fish on swimming velocity, three cases with a body contraction length of ΔL = 0 mm, ΔL = 10 mm, and ΔL = 20 mm are considered in the conducted experiments. In total, the average swimming velocities under 165 situations are measured. The experimental results are presented in Figure 7, which compares the swimming velocity under different frequencies and amplitudes. In the experiments, the same control parameters of frequency are set to the robotic fish. However, the actual frequency of each case is measured, which has some little differences.

**Figure 8:**
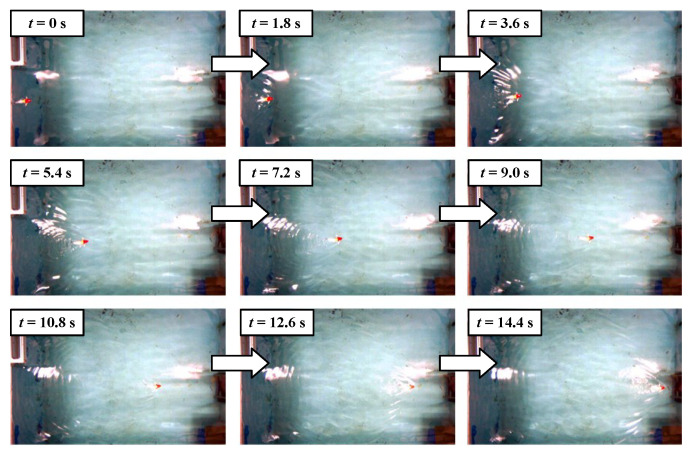
Swimming snapshot sequence of the robotic fish with a maximum speed ( ΔL = 10 mm).

As illustrated in Figure 7, it is evident that the oscillation frequency and amplitude play significant roles in the swimming velocity. The increase in oscillation amplitude leads to a corresponding rise in swimming velocity while augmenting the frequency exhibits an overall increasing trend. However, for each case, there are slight declines observed within the intermediate range of frequencies. As presented in Figure 4, due to limitations imposed by the employed servo motor, higher oscillation frequencies result in reduced actual amplitudes of the tensegrity body’s oscillations, consequently causing a minor decrease in swimming velocity. After that, with the increase in frequency, the velocity will further increase. In these experiments, the maximum swimming velocities of each contraction length are 191 mm/s (0.53 BL/s, ΔL = 0 mm), 295 mm/s (0.84 BL/s, ΔL = 10 mm), and 281 mm/s (0.83 BL/s, ΔL = 20 mm), which are all obtained at the maximum oscillation frequencies (about 2.75 Hz) and amplitudes ( $A=65^{{}^{\circ}}$ ). The snapshot sequences of the swimming motion with maximum velocity are presented in Figure 8. The swimming experimental videos are provided in the Supplementary Materials.

To better analyze the influence of stiffness variation (contraction length) on swimming velocity, we also compare the swimming velocities under identical oscillation amplitudes. As an illustration, Figure 7d presents the comparisons at a rotation amplitude of $65^{{}^{\circ}}$ . We can also find that when the contraction length changes from 0 mm to 10 mm, the swimming velocities all increase in each situation. However, with further increases in contraction length, the swimming velocity changes a little. Even at higher frequencies, the swimming velocity decreases a little. The bending characteristic of the tensegrity body significantly affects the swimming performance. The oscillation amplitude in the air has been explored and the results presented in Figure 4b indicate that the oscillation amplitude of the body with a contraction length of 10 mm is larger than that of the body with a contraction length of 20 mm, which can explain this phenomenon to some extent. In addition, during swimming motion, hydrodynamic forces acting on the body also change the bending characteristic. When the contraction length increases to 20 mm, the stiffness of the body is very large. Therefore, hydrodynamic forces are not large enough to further affect the body bending.

### 4.3. Steering Motion Testing

**Figure 9:**
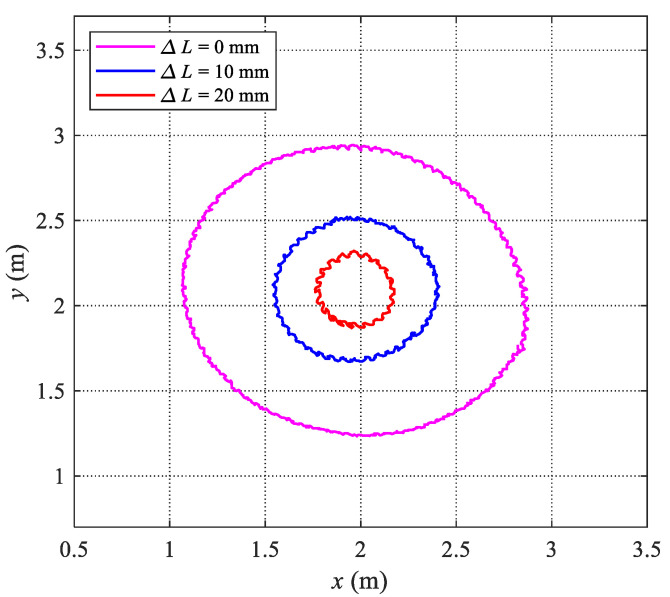
Steering motion trajectories of the tensegrity robotic fish under three body lengths.

**Figure 10:**
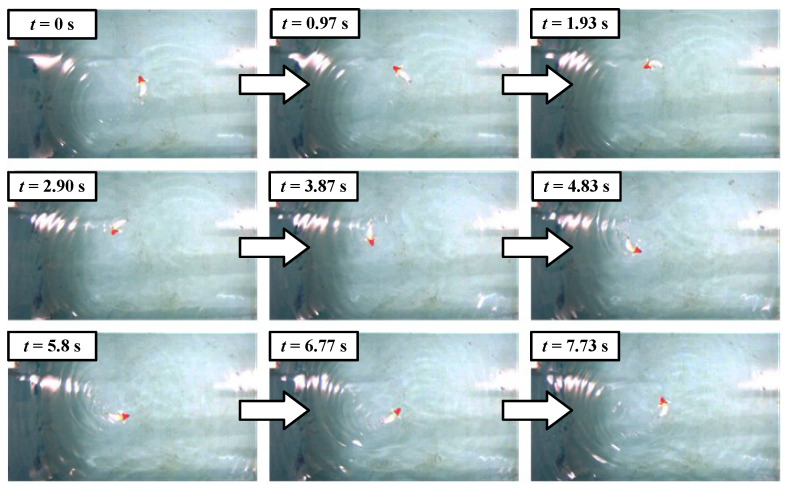
Steering motion sequence of the robotic fish with the minimum radius.

As for the existing tensegrity robotic fish, the maneuverability is usually less considered. To realize the asymmetric undulation propulsion of the tensegrity body, two servo motors rotate in a sinusoidal form with an offset, namely, Asin(2πft+φ)+B . When the offset angle $B\neq 0^{{}^{\circ}}$ , the oscillation of the body is asymmetrical and a steering motion can be performed. During the steering motion testing, the procedure has been preset to the controller of our robotic fish. We only need to send the parameters (*A*, *f*, ϕ , and *B*) to the robotic fish by a wireless module once. The robotic fish will perform the corresponding steering motion automatically. We are mainly concerned about the turning radius and the average angular velocity of the designed tensegrity robotic fish. These indicators are determined by the oscillation frequency and the offset of the oscillation. Therefore, in the steering motion experiments, the maximum oscillation frequencies and maximum offsets for the tensegrity robotic fish with three contraction lengths are given, and the steering motions are recorded by a global camera. As illustrated in Figure 9, these steering motion trajectories are obtained. Furthermore, the turning periods and average angular velocity are also measured. The detailed steering motion performance is tabulated in Table 3. As for the three cases, the maximum steering radius of 900 mm (2.5 BL) and the minimum angular velocity of 10.8°/s are all obtained by the robotic fish with a contraction length of 0 mm. The maximum angular velocity and minimum radius are 46.6°/s and 230 mm (0.68 BL), respectively, which are all obtained by the tensegrity robotic fish with a contraction length of 20 mm. The corresponding steering motion sequences are presented in Figure 10. The steering experimental videos are also provided in the Supplementary Materials. Furthermore, our findings indicate that while swimming velocity is minimally affected by body contraction length ( ΔL= 10 mm or ΔL= 20 mm), there is a significant influence on steering motion.

## 5. Discussion

In this paper, we propose a tensegrity robotic fish and conduct extensive analyses. The main objective of the mathematical model-based analysis is to validate the feasibility of using a tensegrity-based body to achieve fish-like bending motion by driving the wires. To simplify the analysis, we consider only the geometrical relationship in a two-dimensional plane and assume equal length variation in each tensegrity unit. Simulation results demonstrate that the tensegrity body can successfully perform bending motion with wire variations. However, during our oscillation motion testing experiment, there may be differences in the bending behavior of the tensegrity body due to elastic components which significantly influence its properties. In future work, we will establish an accurate model incorporating these elastic components to guide the optimization design of the tensegrity structure.

Regarding the proposed tensegrity body, adjusting the contraction length of wires leads to a corresponding change in the pre-tensions of elastic elements within the tensegrity system, thereby exhibiting a property of stiffness variation. Importantly, our robotic fish equipped with two servo motors for wire-driven mode can easily achieve online stiffness adjustment, offering a novel approach to variable stiffness mechanisms. However, due to the intricate elasticity network present in tensegrity structures, accurately describing the relationship between body stiffness and length variation becomes challenging, and precise stiffness adjustment remains arduous. Furthermore, as a consequence of stiffness variation, changes in body length occur concurrently with variations in bending properties. These factors result in complex coupling effects on swimming performance exhibited by the tensegrity robotic fish. For instance, during swimming performance testing, an increase in body stiffness has less impact on swimming velocity but significantly improves turning radius and angular velocity. Therefore, predicting performance for this proposed tensegrity robotic fish poses considerable challenges. In addition, in this paper, we mainly focus on the realization of stiffness variation with tensegrity structure. Biology inspires not only the design of functional structures but also the modulation strategies, such as the stiffness modification strategy [37], in future works, the optimization of performance with bioinspired stiffness modification strategy will be considered.

## 6. Conclusions

In this paper, we have described a bio-inspired and tensegrity structure-based approach for designing a novel robotic fish that exhibits continuum characteristics and stiffness variation. Drawing inspiration from biological fish with BCF propulsion mode, we propose a simple and easily fabricable continuum robotic fish body composed of modular tensegrity units. A mathematical model is built to analyze the bending properties of the tensegrity structure, demonstrating the fish-like oscillation motion. Additionally, we conduct oscillation motion and load testing under different contraction lengths to observe body stiffness variation characteristics. Based on our validated tensegrity body design, a proof-of-concept bionic robotic fish with a rigid head, a continuum tensegrity body, and a flexible tail was constructed. Extensive experiments with different control parameters were performed to explore the swimming performance of the bionic tensegrity robotic fish. By adjusting the actuation mode of the servo motor, our robotic fish achieves straight swimming motion as well as steering motion. Furthermore, three contraction lengths are considered to analyze the effects of body stiffness on swimming performance. These results validate the effectiveness of the proposed scheme for designing a tensegrity-based robotic fish. Finally, our robotic fish can achieve a maximum swimming speed of 0.84 BL/s at a frequency of 2.75 Hz and a steering motion with a radius of 0.68 BL and an angular velocity of 46.6°/s. In this paper, the proposed bionic design scheme with tensegrity structure presents great potential in designing a novel continuum robotic fish with stiffness variation capability.

In future research, we will further enhance the design of tensegrity robotic fish to achieve better variable stiffness capability and swimming performance. Furthermore, we will also investigate the optimization of stiffness distribution in different tensegrity modules using both theoretical and experimental approaches.

## Figures and Tables

**Table 1:** Physical properties of the tensegrity body.

Components	Length (mm)	Diameter (mm)	Numbers
Longitudinal rod	34	4.5	16
Vertical rod	25	–	6
Transverse spring	15	5	3
Longitudinal spring	20	5	16
Wire	135	1	4

**Table 2:** Technical specifications of the tensegrity robotic fish prototype.

Items	Characteristics
Total mass	835 g
Total length	360 mm
Body length	135 mm
Motor	Servo motor × 2
Controller	STM32F407VGT6, 168 MHz
Power supply	7.4 V rechargeable batteries
Communication unit	Wireless (RF200, 433 MHz)

**Table 3:** Steering motion performance of the tensegrity robotic fish.

Contraction Length	Radius (mm)	Period (s)	Angular Velocity (°/s)
ΔL = 0 mm	900	33.4	10.8
ΔL = 10 mm	440	16.3	22.1
ΔL = 20 mm	230	7.73	46.6

## Data Availability

The data generated during the current study are available from the corresponding author upon reasonable request.

## Supplementary Materials

The following supporting information can be downloaded at: www.mdpi.com/xxx/s1, Video S1: Simulations and experiments of the tensegrity robotic fish.

## References

[1] Fish F.E. (2013). Advantages of natural propulsive systems. Mar. Technol. Soc. J..

[2] Katzschmann R.K., DelPreto J., MacCurdy R., Rus D. (2018). Exploration of underwater life with an acoustically controlled soft robotic fish. Sci. Robot..

[3] Bianchi G., Maffi L., Tealdi M., Cinquemani S.A. (2023). Bioinspired cownose ray robot for seabed exploration. Biomimetics.

[4] Li G., Chen X., Zhou F., Liang Y., Xiao Y., Cao X., Zhang Z., Zhang M., Wu B., Yin S. (2021). Self-powered soft robot in the Mariana Trench. Nature.

[5] Tan X. (2011). Autonomous robotic fish as mobile sensor platforms: Challenges and potential solutions. Mar. Technol. Soc. J..

[6] Sfakiotakis M., Lane D.M., Davies J.B.C. (1999). Review of fish swimming modes for aquatic locomotion. IEEE J. Ocean Eng..

[7] Cui Z., Jiang H. (2016). Design, analysis, and simulation of a planar serial-parallel mechanism for a compliant robotic fish with variable stiffness. Adv. Mech. Eng..

[8] Quinn D., Lauder G.V. (2021). Tunable stiffness in fish robotics: Mechanisms and advantages. Bioinspir. Biomim..

[9] Zhong Q., Zhu J., Fish F.E., Kerr S.J., Downs A.M., Bart-Smith H., Quinn D.B. (2021). Tunable stiffness enables fast and efficient swimming in fish-like robots. Sci. Robot..

[10] Chen D., Xiong Y., Wang B., Tong R., Meng Y., Yu J. (2023). Performance optimization for bionic robotic dolphin with active variable stiffness control. Biomimetics.

[11] Liu Y., Jiang H. (2022). Research development on fish swimming. Chin. J. Mech. Eng..

[12] Raj A., Thakur A. (2016). Fish-inspired robots: Design, sensing, actuation, and autonomy—A review of research. Bioinspir. Biomim..

[13] Chen B., Jiang H. (2019). Swimming performance of a tensegrity robotic fish. Soft Robot..

[14] Chen D., Wu Z., Meng Y., Tan M., Yu J. (2022). Development of a high-speed swimming robot with the capability of fish-like leaping. IEEE/ASME Trans. Mechatron..

[15] Xu M., Yu Y. (2023). Effects of body stiffness on propulsion during fish self-propelled swimming. Phys. Fluids.

[16] Tytell E.D., Leftwich M.C., Hsu C. (2016). Role of body stiffness in undulatory swimming: Insights from robotic and computational models. Phys. Rev. Fluids.

[17] Zheng C., Ding J., Dong B., Lian G., He K., Xie F. (2022). How non-uniform stiffness affects the propulsion performance of a biomimetic robotic fish. Biomimetics.

[18] Chen D., Wu Z., Dong H., Tan M., Yu J. (2020). Exploration of swimming performance for a biomimetic multi-joint robotic fish with a compliant passive joint. Bioinspir. Biomim..

[19] White C.H., Lauder G.V., Bart-Smith H. (2021). Tunabot Flex: A tuna-inspired robot with body flexibility improves high-performance swimming. Bioinspir. Biomim..

[20] Zou Q., Zhou C., Lu B., Liao X., Zhang Z. (2022). Tail-stiffness optimization for a flexible robotic fish. Bioinspir. Biomim..

[21] Park Y.J., Huh T.M., Park D., Cho K.J. (2014). Design of a variable-stiffness flapping mechanism for maximizing the thrust of a bio-inspired underwater robot. Bioinspir. Biomim..

[22] Zhu C., Deng L., Wang X., Yin Z., Zhou C. Design and modeling of elastic variable stiffness robotic fish tail. Proceedings of the IEEE International Conference on Mechatronics and Automation.

[23] Reksowardojo A.P., Senatore G. (2023). Design of ultra-lightweight and energy-efficient civil structures through shape morphing. Comput. Struct..

[24] Wang Q., Senatore G., Jansen K., Habraken A., Teuffel P. (2022). Multi-scale experimental testing on variable stiffness and damping components for semi-active structural control. Comput. Struct..

[25] Van den Berg S.C., Scharff R.B.N., Rusák Z., Wu J. (2022). OpenFish: Biomimetic design of a soft robotic fish for high speed locomotion. HardwareX.

[26] Mitin I., Korotaev R., Ermolaev A., Mironov V., Lobov S.A., Kazantsev V.B. (2022). Bioinspired propulsion system for a thunniform robotic fish. Biomimetics.

[27] Li K., Jiang H., Wang S., Yu J. (2018). A soft robotic fish with variable-stiffness decoupled mechanisms. J. Bionic Eng..

[28] Jusufi A., Vogt D.M., Wood R.J., Lauder G.V. (2017). Undulatory swimming performance and body stiffness modulation in a soft robotic fish-inspired physical model. Soft Robot..

[29] Qiu C., Wu Z., Wang J., Tan M., Yu J. (2023). Locomotion optimization of a tendon-driven robotic fish with variable passive tail fin. IEEE Trans. Ind. Electron..

[30] Kwak B., Choi B., Bae J. (2023). Development of a stiffness-adjustable articulated paddle and its application to a swimming robot. Adv. Intell. Syst..

[31] Zappetti D., Arandes R., Ajanic E., Floreano D. (2020). Variable-stiffness tensegrity spine. Smart Mater. Struct..

[32] Zappetti D., Jeong S.H., Shintake J., Floreano D. (2020). Phase changing materials-based variable-stiffness tensegrity structures. Soft Robot..

[33] Zhang J., Wang B., Chen H., Bai J., Wu Z., Liu J., Peng H., Wu J. (2023). Bioinspired continuum robots with programmable stiffness by harnessing phase change materials. Adv. Mater. Technol..

[34] Bliss T., Iwasaki T., Bart-Smith H. (2013). Central pattern generator control of a tensegrity swimmer. IEEE/ASME Trans. Mechatron..

[35] Shintake J., Zappetti D., Peter T., Ikemoto Y., Floreano D. Bio-inspired tensegrity fish robot. Proceedings of the 2020 IEEE International Conference on Robotics and Automation (ICRA).

[36] Chen B., Jiang H. (2021). Body stiffness variation of a tensegrity robotic fish using antagonistic stiffness in a kinematically singular configuration. IEEE Trans. Robot..

[37] Kiakojouri F., De Biagi V., Abbracciavento L. (2023). Design for robustness: Bio-inspired perspectives in structural engineering. Biomimetics.

